# Federated Learning in Small-Cell Networks: Stochastic Geometry-Based Analysis on the Required Base Station Density

**DOI:** 10.3390/s23167184

**Published:** 2023-08-15

**Authors:** Khoa Anh Nguyen, Quan Anh Nguyen, Jun-Pyo Hong

**Affiliations:** 1 Department of Information and Communications Engineering, Pukyong National University, Busan 48513, Republic of Korea; khoana@pukyong.ac.kr; 2 Hello Health Group, Singapore 079333, Singapore; quan.nguyen@hellohealthgroup.com

**Keywords:** federated learning, small-cell networks, stochastic geometry, base station density, Poisson point process

## Abstract

Recently, federated learning (FL) has been receiving great attention as an effective machine learning method to avoid the security issue in raw data collection, as well as to distribute the computing load to edge devices. However, even though wireless communication is an essential component for implementing FL in edge networks, there have been few works that analyze the effect of wireless networks on FL. In this paper, we investigate FL in small-cell networks where multiple base stations (BSs) and users are located according to a homogeneous Poisson point process (PPP) with different densities. We comprehensively analyze the effects of geographic node deployment on the model aggregation in FL on the basis of stochastic geometry-based analysis. We derive the closed-form expressions of coverage probability with tractable approximations and discuss the minimum required BS density for achieving a target model aggregation rate in small-cell networks. Our analysis and simulation results provide insightful information for understanding the behaviors of FL in small-cell networks; these can be exploited as a guideline for designing the network facilitating wireless FL.

## 1. Introduction

Recent advances in the sensing and computation capabilities of mobile devices make it possible for end-users to generate various types of data and exploit these data for autonomous and intelligent services [1]. Machine learning (ML), which builds a mathematical model based on training data to make predictions or decisions without human intervention [2], can be considered an essential building block for embedding artificial intelligence (AI) within services.

Traditional ML technologies depend on the prerequisite that the private data on edge devices should be collected at a central parameter server for training the model. However, this centralized approach has to bear the risk of personal data exposure and can violate privacy regulations, which are becoming progressively more stringent over time [3]. This also leads to immense communication overheads for data transmission, causing intolerable latency and communication resource inefficiency [4].

To deal with the limitations of traditional centralized ML, federated learning (FL) has emerged. FL adopts a distributed training approach, where each edge device trains a common model with its own local data samples in a distributed manner and forwards the locally trained model to the parameter server for subsequent operations [5]. Accordingly, by decoupling model training from the necessity of private data collection, the FL mechanism enables users to exploit ML models trained with enormous data without severely compromising user security and privacy threats, as well as communication costs, making this option relevant for numerous wireless applications [6].

To fulfill stringent performance requirements, wireless communication networks have evolved in a complicated manner. In particular, the emergence of small-cell networks made it harder to optimize network performance with inter-cell interference [4]. Thus, FL in small-cell networks are not well understood, triggering challenges for FL implementation in cellular settings [6]. Many previous studies propose methods to address the challenges of FL performance in wireless communications.

Amongst the primary challenges, communication efficiency is a critical training bottleneck due to updates’ high dimensionality, massive quantities of devices, or unreliability of devices’ network conditions. To alleviate this issue, importance-based updating schemes have been studied in [7,8]. The edge stochastic gradient descent (eSGD) algorithm in [7] assigns only a fraction of the gradients for the model update based on the loss values at two successive training iterations, which helps to save a significant fraction of communication resources. In [8], a communication-mitigated federated learning (CMFL) algorithm has been proposed to compare a participant’s local update with the global value to evaluate the update’s relevance, and the irrelevant updates are eliminated to improve accuracy. To reduce the reporting data size, local update compression has been considered in [9,10]. With parameter pruning, trained quantization, and Huffman coding, it has been shown that the the size of a bwell-known model can be significantly reduced without loss of accuracy [9]. In addition to reducing model size with compression, federated dropout, which allows users to train only a subset of model, has been proposed to not only improve the communication efficiency but also reduce the local computation.

Notably, over-the-air computation (AirComp) has received attention as an alternative approach for communication-efficient FL by leveraging the superposition property of a multiple-access channel. Specifically, if multiple devices transmit their analog-modulated local update signals over the same communication resource, the fusion center (FC) can obtain the averaged local updates from its received signal without additional computation at FC. When the number of participating devices is large, AirComp-based FL has been shown to outperform traditional digital communication-based FL in terms of the number of channel users necessary for model convergence [11,12,13].

Although there have been several works that address challenges of incorporating federated learning into wireless networks, FL over a cellular network with multiple base stations (BSs) and devices has not yet been investigated while taking account into geographic deployment. The effect of geographic deployment of BSs and users can be well described on the basis of stochastic geometry-based analysis with the assumption of a Poisson point process (PPP) [14,15]. Although conventional work on stochastic geometry-based cellular network analysis has provided meaningful information for understanding cellular networks by providing closed-form solutions, most analysis results cannot directly apply to FL scenarios due to some assumptions that are required for general cellular communication. For example, some have assumed that each BS serves only one user with a given resource block at a time and that there is at least one user within a Voronoi cell. Several papers [16,17,18] have explored the topic of FL with multi-user association. They have focused on proposing new communication architectures to reduce the communication overhead for FL, called hierarchical FL, in small-scale cellular networks. Even though they have proposed new communication architectures for communication-efficient FL, it is hard to directly apply them to existing cellular networks with distance-based user association and inter-cell interference. Furthermore, their performance analysis results do not easily offer insights into large-scale cellular networks, especially when considering the geographic deployment of nodes.

Motivated by these, we investigated multi-user association-based model aggregation for FL in large-scale cellular networks. Our work focused on analyzing the model aggregation performance and optimizing certain system parameters for communication-efficient FL within existing cellular networks, rather than proposing a new communication strategy. Based on stochastic geometry-based analysis, we derived the closed-form expressions of coverage probability with tractable approximations. With the closed-form expressions, we derived the minimum required BS density for achieving a target model aggregation rate by using a two-step iterative method. The proposed algorithm is helpful to reduce the time and spectrum resources consumed for FL in large-scale cellular networks. Simulation results validated that the coverage probability expressions obtained with stochastic geometry-based analysis describe the actual coverage probability well. Furthermore, simulation results showed the effects of system parameters on the optimized transmission rate and BS density for achieving the target model aggregation performance. Our analysis and simulation results with discussions on the model aggregation rate are valuable for providing insightful information for understanding the behaviors of FL in large-scale cellular networks. They can also be exploited as guidelines for designing cellular networks that facilitate wireless FL.

Considering the aforementioned motivations, we summarize the contributions of this paper below:

1. To the best of our knowledge, this is the first work that takes the geometry of wireless communication network into account in analyzing FL performance, that analyzes the model aggregation performance, and that optimizes certain system parameters for communication-efficient FL within existing cellular networks;
2. Based on the stochastic geometry framework, we derive the closed-form expressions of the approximated coverage probability for some special cases in small-cell networks where each base station is capable of receiving updates from multiple associated devices with orthogonal spectrum allocation;
3. With the closed-form expressions, we propose an iterative algorithm to optimize communication parameters for achieving a target model aggregation rate which can be helpful to reduce the time and spectrum resources consumed for FL in large-scale cellular networks. The purpose is to minimize the communication latency in model aggregation of FL under the existing cellular network with distance-based user association;
4. Analysis and simulation results provide insightful information for understanding the behaviors of FL over large-scale small-cell networks and provide a guideline for designing cellular networks which facilitate wireless FL.

The remaining sections in this paper are organized as follows. Section 2 describes the system model, discusses the key network parameters of small-cell network, and formulates optimization problems for two different FL scenarios. Section 3 derives the closed-form expressions of coverage probability in small-cell networks. Based on the coverage expressions, we derive solutions to the optimization problems with the iterative method in Section 4. Section 5 validates analysis results through extensive simulations. Finally, Section 6 concludes the paper.

## 2. System Model

**Figure 1:**
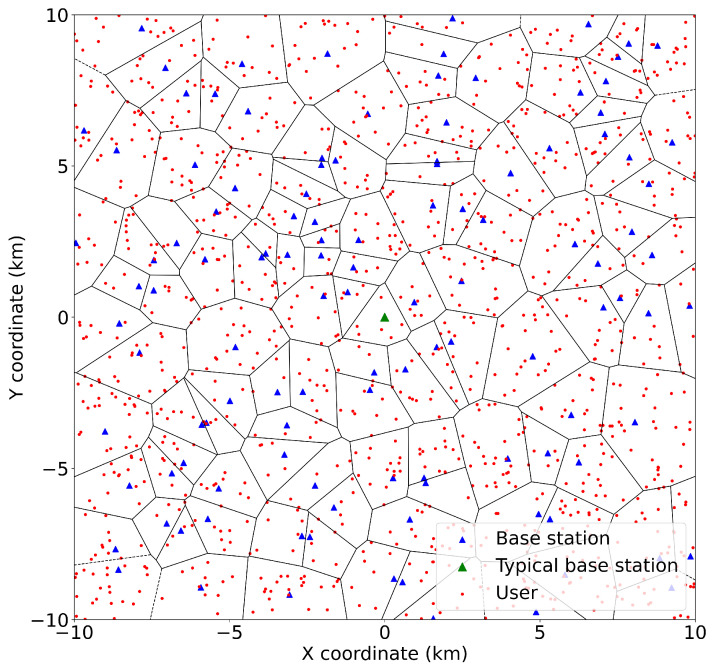
Deployments of BSs and users in an area measuring 20km×20km.

We consider FL in small-cell networks, where the FC updates the global model by combining the locally updated models delivered from users through BSs to the FC. The links between BSs and FC are assumed to have infinite capacity within their wired backhaul connections. BSs and users are assumed to be located in the Euclidean plane, according to homogeneous PPPs $\Phi_{\mathrm{BS}}$ with density $\lambda_{\mathrm{BS}}$ and $\Phi_{\mathrm{UE}}$ with density $\lambda_{\mathrm{UE}}$. This assumption makes the network performance analysis significantly more tractable than the traditional grid-based analysis, without causing significant error in the network performance [15]. Each user is assumed to be associated with its nearest BS, so that each BS associates with all users located in its Voronoi cell. An example of BS and user deployment is illustrated in Figure 1.

The procedures of FL in small-cell networks can be summarized as follows:

1. In the beginning of update round *t*, FC broadcasts the parameters of the global model, $w^{\left(t\right)}\in R^{D}$, to the distributed users via BSs. Since BSs deliver the same signal $w^{\left(t\right)}$, all users are assumed to successfully receive $w^{\left(t\right)}$ without interference;
2. Each user updates the model parameters on the basis of its local dataset and transmits its local update model to the associated BS. If there are $K_{i}$ users associated with BS, $i\in \Phi_{\mathrm{BS}}$, each associated user transmits its local update model with the bandwidth $\frac{B}{K_{i}}$, where *B* denotes total bandwidth;
3. Each BS averages out the local updates received from its associated users and forwards it to FC;
4. FC updates the global model by combining the parameters aggregated from BSs. Then, the method proceeds to update round t+1 by starting from step 1 if the convergence condition is not satisfied.

Since the local update report from a user to its associated BS suffers from inter-cell interference and limited communication resources, step 2 can easily become a bottleneck for FL in small-cell networks. For this reason, we focus on step 2 to facilitate FL in small-cell networks.

Following Slivnyak’s theorem [19], all analysis is conducted for a typical BS located at the origin $o\in \Phi_{\mathrm{BS}}$. The received signal of the typical BS can be represented as

(1) $$\begin{array}{c} y_{o}=h_{j}r_{j}^{-\alpha /2}x_{j}+\sum_{j^{\prime }\in \Phi_{o,\inf}}h_{j^{\prime }}r_{j^{\prime }}^{-\alpha /2}x_{j^{\prime }}+w, \end{array}$$

where the index *j* represents one of the users associated with the typical BS, $h_{i}∼\mathrm{CN}\left(0,1\right)$ denotes the Rayleigh block fading channel gain of user *i*, $r_{i}$ denotes link distance between user *i* and the typical BS, α denotes path-loss exponent, $x_{i}$ denotes the transmit signal of user *i*, and $w∼\mathrm{CN}(0,\sigma^{2})$ denotes additive noise. All users are assumed to transmit their update with a constant power *P*. The set $\Phi_{o,\inf}$ consists of the other cell users interfering with the signal reception of the BS. The channel state information (CSI) $h_{j}$ is assumed to be available only at the BS.

By treating interference as additive noise, the received signal-to-noise-plus-interference (SINR) of a user can be represented as

(2) $$\begin{array}{cc} \gamma & =\frac{|h_{j}{|}^{2}r_{j}^{-\alpha }P}{\sum_{j^{\prime }\in \Phi_{o},\inf}{\left|h_{j^{\prime }}\right|}^{2}r_{j^{\prime }}^{-\alpha }P+\sigma^{2}}\\ \; & =\frac{g_{j}r_{j}^{-\alpha }}{\sum_{j^{\prime }\in \Phi_{o},\inf}g_{j^{\prime }}r_{j^{\prime }}^{-\alpha }+\sigma^{2}}, \end{array}$$

where $g_{i}={\left|h_{i}\right|}^{2}P$ follows an exponential distribution with mean *P*. For the transmission rate *u*, the conditional coverage probability given k>0 users within the Voronoi cell is defined as

(3) $$\begin{array}{cc} p_{c}\left(k\right) & =\mathrm{Pr}\;\left[\frac{B}{K}\log_{2}\left(1+\gamma \right)\geq u|K=k\right]\\ \; & =\mathrm{Pr}\;\left[\gamma \geq 2^{\frac{uK}{B}}-1|K=k\right]\\ \; & =\mathrm{Pr}\;\left[\gamma \geq T(K)|K=k\right], \end{array}$$

where $T\left(K\right)=2^{\frac{uK}{B}}-1$ denotes an SINR threshold for successful signal reception. Accordingly, for a unit area of model aggregation, the expected number of aggregated bits of locally trained models in a single transmission interval can be represented as

(4) $$\begin{array}{cc} Q & =u\lambda_{\mathrm{UE}}\sum_{k=1}^{\infty }\mathrm{Pr}\;\left[K=k\right]p_{c}\left(k\right)\\ \; & =u\lambda_{\mathrm{UE}}P_{c}, \end{array}$$

where $P_{c}≜\sum_{k=1}^{\infty }\mathrm{Pr}\;\left[K=k\right]p_{c}\left(k\right)$ denotes the coverage probability. As a result, *Q* is directly related to the communication latency in the model aggregation phase of FL. The time and spectrum resources consumed for the successful model aggregation are inversely proportional to *Q*. Since the model aggregation phase is considered as a bottleneck in FL over a wireless network, we believe that improving the model aggregation rate is important to expedite communication-efficient FL. Based on this understanding, we considered the following optimization problems in two different scenarios.

In the first scenario, for given node densities $\lambda_{\mathrm{BS}}$ and $\lambda_{\mathrm{UE}}$, we optimized the transmission rate *u* to maximize the model aggregation rate *Q*. Then, the problem was formulated as

(5) $$\begin{array}{c} \underset{u}{\mathrm{maximize}}\;Q. \end{array}$$

In the second scenario, we optimized $\lambda_{\mathrm{BS}}$ as well as *u* to find the minimum required BS deployment for achieving a target aggregation rate $Q_{\mathrm{target}}$. Then, the problem could be formulated as

(6) $$\begin{array}{cc} \underset{\lambda_{\mathrm{BS}},\;u}{\mathrm{minimize}} & \;\lambda_{\mathrm{BS}}\\ \mathrm{subject}\;\mathrm{to} & \;Q\geq Q_{\mathrm{target}}. \end{array}$$

## 3. Performance Analysis and Proposed Algorithm

In PPP, the number of users in a typical Voronoi cell, *K*, is dependent on the Voronoi cell size. For a given cell, of size A=a, the number of users *K* follows a Poisson distribution with mean $a\lambda_{\mathrm{UE}}$. Accordingly, the probability mass function (PMF) of *K* can be expanded as

(7) $$\begin{array}{cc} \mathrm{Pr}[K=k]\; & =\int_{0}^{\infty }\mathrm{Pr}\left[K=k|A=a\right]\cdot f_{A}\left(a\right)\;da\\ \; & =\int_{0}^{\infty }\frac{{\left(\lambda_{\mathrm{UE}}a\right)}^{k}}{k!}e^{-\lambda_{\mathrm{UE}}a}\cdot \frac{c^{c}}{\Gamma (c)}\lambda_{\mathrm{BS}}^{c}a^{c-1}e^{-c\lambda_{\mathrm{BS}}a}\;da, \end{array}$$

where Γ(·) denotes the Gamma function, $f_{A}\left(a\right)=\frac{c^{c}}{\Gamma (c)}\lambda_{\mathrm{BS}}^{c}a^{c-1}e^{-c\lambda_{\mathrm{BS}}a}$ denotes the probability density function (PDF) of typical Voronoi cell size [20,21], and c=3.5 is a constant. Thus,

(8) $$\begin{array}{cc} \mathrm{Pr}[K=k]\; & =\frac{c^{c}}{\Gamma (c)}\frac{\lambda_{\mathrm{UE}}^{k}\lambda_{\mathrm{BS}}^{c}}{k!}\int_{0}^{\infty }a^{k+c-1}e^{-(\lambda_{\mathrm{UE}}+c\lambda_{\mathrm{BS}})a}\;da\\ \; & =\frac{c^{c}}{\Gamma (c)}\frac{\lambda_{\mathrm{UE}}^{k}\lambda_{\mathrm{BS}}^{c}}{k!}\frac{\Gamma (k+c)}{{\left(\lambda_{\mathrm{UE}}+c\lambda_{\mathrm{BS}}\right)}^{k+c}}\\ \; & =\frac{c^{c}}{\Gamma (c)}\frac{{\left(\lambda_{\mathrm{BS}}/\lambda_{\mathrm{UE}}\right)}^{c}}{k!}\frac{\Gamma (k+c)}{{\left(1+c\lambda_{\mathrm{BS}}/\lambda_{\mathrm{UE}}\right)}^{k+c}}\\ \; & =\frac{c^{c}}{\Gamma (c)}\frac{r_{\lambda }^{c}}{k!}\frac{\Gamma (k+c)}{{\left(1+cr_{\lambda }\right)}^{k+c}}\\ \; & =\frac{{\left(\frac{cr_{\lambda }}{1+cr_{\lambda }}\right)}^{c}}{\Gamma (c)}\frac{\Gamma (k+c)}{k!{\left(1+cr_{\lambda }\right)}^{k}}, \end{array}$$

where $r_{\lambda }=\frac{\lambda_{\mathrm{BS}}}{\lambda_{\mathrm{UE}}}$. The number of users in the typical Voronoi cell depends on the ratio between BS and user densities.

### 3.1. Coverage Probability

In our uplink system model, the conditional coverage probability can be obtained by modifying the coverage probability expression of the downlink system considered in [15]. The downlink system in [15] assumed there was at least one user in every Voronoi cell, and, therefore, all BSs were active: $\lambda_{\mathrm{BS}}=\lambda_{\mathrm{act}}$. However, such an assumption is valid only when the ratio $r_{\lambda }$ is very low. To derive a general expression that could be applicable to various BS/user deployments, we relaxed the low $r_{\lambda }$ assumption and introduced the active BS density to the coverage probability expression. Furthermore, since our system model assumed that each BS served all its associated users with equal-bandwidth allocation (contrary to the downlink system in [15]), the SINR threshold became a function of random variable *K* in the coverage probability expression. By additionally taking into account the active BS density and random SINR threshold, the conditional coverage probability (3) could be represented as the following lemma.

**Lemma** **1** (Conditional coverage probability)**.** *For a given transmission rate u and* K=k*, the conditional coverage probability* (3) *can be computed as*

(9) $$\begin{array}{cc} p_{c}\left(k\right)\; & =\pi \lambda_{\mathrm{BS}}\int_{0}^{\infty }e^{-\pi \left(\lambda_{\mathrm{BS}}+\lambda_{\mathrm{act}}\rho \left(k\right)\right)v-\frac{1}{P}T\left(k\right)\sigma^{2}v^{\alpha /2}}dv, \end{array}$$

*where*

(10) $$\begin{array}{c} \rho \left(k\right)=T{\left(k\right)}^{2/\alpha }\int_{T{\left(k\right)}^{-2/\alpha }}^{\infty }\frac{1}{1+t^{\alpha /2}}dt, \end{array}$$

*and $\lambda_{\mathrm{act}}$ denotes the density of active BSs that contain at least one user in their Voronoi cell.*

Based on the independence of BSs from user deployments, the deployment of active BSs follows PPP with density

(11) $$\begin{array}{cc} \lambda_{\mathrm{act}} & =\lambda_{\mathrm{BS}}\mathrm{Pr}\left[K\neq 0\right]\\ \; & =\lambda_{\mathrm{BS}}\left(1-{\left(\frac{cr_{\lambda }}{1+cr_{\lambda }}\right)}^{c}\right). \end{array}$$

Then, based on (8) and (9), the coverage probability can be represented as

(12) $$\begin{array}{cc} P_{c} & =\sum_{k=1}^{\infty }p_{c}\left(k\right)\mathrm{Pr}\left[K=k\right]\\ \; & =\frac{\pi (\lambda_{\mathrm{BS}}-\lambda_{\mathrm{act}})}{\Gamma (c)}\sum_{k=1}^{\infty }\frac{\Gamma (k+c)}{k!{\left(1+cr_{\lambda }\right)}^{k}}J\left(k\right), \end{array}$$

where $J\left(k\right)=\int_{0}^{\infty }e^{-\pi \left(\lambda_{\mathrm{BS}}+\lambda_{\mathrm{act}}\rho \left(k\right)\right)v-\frac{1}{P}T\left(k\right)\sigma^{2}v^{\alpha /2}}dv$.

### 3.2. Coverage Probability in High SNR Regime

In a high SNR regime with a large *P*, the effect of additive noise becomes negligible compared to the inter-cell interference. Accordingly, the conditional coverage probability (9) simplifies to

(13) $$\begin{array}{cc} p_{c}\left(k\right) & =\pi \lambda_{\mathrm{BS}}\int_{0}^{\infty }e^{-\pi (\lambda_{\mathrm{BS}}+\lambda_{\mathrm{act}}\rho \left(k\right))v}dv\\ \; & =\pi \lambda_{\mathrm{BS}}\frac{1}{\pi (\lambda_{\mathrm{BS}}+\lambda_{\mathrm{act}}\rho \left(k\right))}\\ \; & =\frac{1}{1+\mathrm{Pr}[K\neq 0]\rho (k)}. \end{array}$$

Then, the coverage probability (12) can be reduced to

(14) $$\begin{array}{c} P_{c}=\frac{\mathrm{Pr}[K=0]}{\Gamma (c)}\sum_{k=1}^{\infty }\frac{\Gamma (k+c)}{k!{\left(1+cr_{\lambda }\right)}^{k}}\frac{1}{1+\mathrm{Pr}[K\neq 0]\rho (k)}. \end{array}$$

**Remark** **1.** *In a high SNR regime, the following emerge:*

- *The coverage probability* (14) *is dependent on the densities of BSs and users, since the probabilities* Pr[K=0] *and* Pr[K≠0] *are functions of* $r_{\lambda }$*. This observation is different from the coverage probability expression presented in [15];*
- *The conditional coverage probability is a decreasing function of the user density* $\lambda_{\mathrm{UE}}$*, given*  K=k *and*  $\lambda_{\mathrm{BS}}$*, and it is bounded below by*
  (15) $$\begin{array}{cc} p_{c}\left(k\right) & \geq \lim_{r_{\lambda }\to 0}p_{c}\left(k\right)\\ \; & =\frac{1}{1+\rho (k)}. \end{array}$$

**Special** **Case:**  α=4

For a path-loss exponent α=4, Equation (10) simplifies to

(16) $$\begin{array}{cc} \rho (k) & =\sqrt{T(k)}\int_{{\sqrt{T(k)}}^{-1}}^{\infty }\frac{1}{1+t^{2}}dt\\ \; & =\sqrt{T(k)}\arctan\;\left(\;\sqrt{T(k)}\right). \end{array}$$

Then, the coverage probability (14) reduces to

(17) $$\begin{array}{cc} P_{c} & =\frac{\mathrm{Pr}[K=0]}{\Gamma (c)}\sum_{k=1}^{\infty }\frac{\Gamma (k+c)}{k!{\left(1+cr_{\lambda }\right)}^{k}}\\ \; & \;\;\;\times \frac{1}{1+\mathrm{Pr}\left[K\neq 0\right]\sqrt{T(k)}\arctan\;\left(\;\sqrt{T(k)}\right)}. \end{array}$$

Even though the coverage probability is simplified with the assumption α=4, its expression (17) is still complicated to handle. Hence, instead of computing exact coverage probability $P_{c}$ by taking the expectation of $p_{c}\left(k\right)$ over the random variable *K*, we proposed to use its approximation $p_{c}\left(E\left[K\right]\right)$. This approximation is validated later in the simulation results in Section 6.

The expected number of users in a Voronoi cell can be obtained by

(18) $$\begin{array}{cc} E[K] & =\lambda_{\mathrm{UE}}S\left(1,1\right)E\left[A\right]\\ \; & =\lambda_{\mathrm{UE}}\int_{0}^{\infty }af_{A}\left(a\right)\;da\\ \; & =\frac{\lambda_{\mathrm{UE}}}{\lambda_{\mathrm{BS}}}=\frac{1}{r_{\lambda }}, \end{array}$$

where S(n,k) denotes the Stirling number of the second kind. Eventually, for high SNR and α=4, the approximated coverage probability is represented by

(19) $$\begin{array}{cc} P_{c} & \approx p_{c}\left(E[K]\right)\\ \; & =\frac{1}{1+\left(1-{\left(\frac{cr_{\lambda }}{1+cr_{\lambda }}\right)}^{c}\right)\;\sqrt{2^{u/Br_{\lambda }}-1}\arctan\;\left(\sqrt{2^{u/Br_{\lambda }}-1}\right)}. \end{array}$$

## 4. Transmission Rate and BS Density Optimization

In the first scenario, the optimization problem (5) is equivalent to the problem maximizing the throughput $uP_{c}$ with respect to the transmission rate *u*. Accordingly, in the high SNR regime with α=4, based on (19), the optimization problem can be re-written by

(20) $$\begin{array}{c} \underset{u}{\mathrm{maximize}}\;f\left(u,\lambda_{\mathrm{BS}}\right), \end{array}$$

where

(21) $$\begin{array}{cc} \; & f(u,\lambda_{\mathrm{BS}})\\ \; & ≜\frac{u}{1\;+\;\left(\;1\;-\;{\left(\frac{c\lambda_{\mathrm{BS}}}{\lambda_{\mathrm{UE}}+c\lambda_{\mathrm{BS}}}\right)}^{c}\right)\;\sqrt{2^{\frac{u\lambda_{\mathrm{UE}}}{B\lambda_{\mathrm{BS}}}}\;-\;1}\arctan\;\left(\;\sqrt{2^{\frac{u\lambda_{\mathrm{UE}}}{B\lambda_{\mathrm{BS}}}}\;-\;1}\right)}. \end{array}$$

Experimentally, the objective $f\left(u,\lambda_{\mathrm{BS}}\right)=Q/\lambda_{\mathrm{UE}}$ is a continuous bell-shaped function with respect to the transmission rate *u*. Based on this observation, we can see that the solution of problem (20) satisfies the condition as follows:

(22) $$\begin{array}{cc} \; & \frac{\partial }{\partial u}f\left(u^{†},\lambda_{\mathrm{BS}}\right)=\frac{1}{u}f\left(u,\lambda_{\mathrm{BS}}\right)(1-f\left(u,\lambda_{\mathrm{BS}}\right)\\ \; & \;\;\;\times \left(\frac{CD\log2-2^{Dx-1}\arctan\;\left(\sqrt{2^{Dx}-1}\right)}{\sqrt{2^{Dx}-1}}+\frac{1}{2}CD\log2\right))\\ \; & =0, \end{array}$$

where $C≜1-\left(\frac{c\lambda_{\mathrm{BS}}}{\lambda_{\mathrm{UE}}+c\lambda_{\mathrm{BS}}}\right)$ and $D≜\frac{\lambda_{\mathrm{UE}}}{B\lambda_{\mathrm{BS}}}$. Then, to find the solution $u^{†}$ that satisfies condition (22), we can apply the bisection method. Based on Algorithm 1, the solution to problem (20) is obtained as

(23) $$\begin{array}{c} u^{†}=BISECTION\left(f_{1}\left(u\right),\;u_{\min},\;u_{\max},\;0,\;\epsilon_{1}\right), \end{array}$$

where $f_{1}\left(u\right)=\frac{\partial }{\partial u}f\left(u,\lambda_{\mathrm{BS}}\right)$, $\epsilon_{1}>0$ denotes an arbitrary small constant, and $u_{\min}$ and $u_{\max}$ denote the minimum and maximum values of the transmission rate, respectively.

In the second scenario, we adopted a two-step iterative method to solve the joint optimization problem (6). According to (21), for a given transmission rate *u*, the model aggregation rate *Q* is a monotonically increasing function of the BS density $\lambda_{\mathrm{BS}}$. Hence, the minimum required BS density $\lambda_{\mathrm{BS}}^{†}$ for a given *u* satisfies the constraint with the equality

(24) $$\begin{array}{c} \lambda_{\mathrm{UE}}f\;\left(u,\lambda_{\mathrm{BS}}^{†}\right)=Q_{\mathrm{target}}. \end{array}$$

Based on (24), the density $\lambda_{\mathrm{BS}}^{†}$ can be obtained by

(25) $$\begin{array}{c} \lambda_{\mathrm{BS}}^{†}=BISECTION\;\left(f_{2}\left(\lambda_{\mathrm{BS}}\right),\;\lambda_{\mathrm{BS},\min},\;\lambda_{\mathrm{BS},\max},\;Q_{\mathrm{target}}/\lambda_{\mathrm{UE}},\;\epsilon_{2}\right), \end{array}$$

where $f_{2}\left(\lambda_{\mathrm{BS}}\right)$ is equivalent to the function $f(u,\lambda_{\mathrm{BS}})$ with a fixed value of *u*, $\epsilon_{2}>0$ denotes an arbitrary small constant, and $\lambda_{\mathrm{BS},\min}$ and $\lambda_{\mathrm{BS},\max}$ denote the minimum and maximum values of the BS density, respectively. Eventually, the solution to problem (6) can be obtained by alternately solving Equations (22) and (24) with Algorithm 1. The process of solving problem (6) is summarized by Algorithm 2.
**Algorithm 1** Bisection Method.1:**function** Bisection($f,x_{\mathrm{low}},\;x_{\mathrm{high}},\;s,\;\epsilon$)2:    **while** $x_{\mathrm{high}}-x_{\mathrm{low}}>\epsilon$ **do**3:        $x_{\mathrm{temp}}\leftarrow \left(x_{\mathrm{low}}+x_{\mathrm{high}}\right)/2$4:        **if** $\left(f\;\left(x_{\mathrm{low}}\right)-s\right)\left(f\;\left(x_{\mathrm{temp}}\right)-s\right)<0$ **then**5:           $x_{\mathrm{high}}\leftarrow x_{\mathrm{temp}}$6:        **else**7:           $x_{\mathrm{low}}\leftarrow x_{\mathrm{temp}}$8:        **end if**9:    **end while**10:   **return** $x_{\mathrm{temp}}$11:**end function**

**Algorithm 2** Two-Step Iterative Method.
1:Initialize $u_{\min},\;u_{\max},\;\lambda_{\mathrm{BS},\min},\;\lambda_{\mathrm{BS},\max}$2:Initialize $\epsilon_{1},\;\epsilon_{2}$3:Initialize $u_{\mathrm{new}}\leftarrow u_{\max}$4:Initialize $\lambda_{\mathrm{BS},\mathrm{new}}\leftarrow \lambda_{\mathrm{BS},\max}$5:
**repeat**
6:     $u_{\mathrm{temp}}\leftarrow u_{\mathrm{new}}$7:     $\lambda_{\mathrm{BS},\mathrm{temp}}\leftarrow \lambda_{\mathrm{BS},\mathrm{new}}$8:     Set $f_{1}\left(u\right)=\frac{\partial }{\partial u}f\left(u,\;\lambda_{\mathrm{BS},\mathrm{temp}}\right)$9:     $u_{\mathrm{new}}\leftarrow \mathrm{BISECTION}\left(f_{1}\left(u\right),\;u_{\min},\;u_{\max},\;0,\;\epsilon_{1}\right)$10:    Set $f_{2}\left(\lambda_{\mathrm{BS}}\right)=f\left(u_{\mathrm{new}},\lambda_{\mathrm{BS}}\right)$11:    $\lambda_{\mathrm{new}}\leftarrow \mathrm{BISECTION}\left(f_{2}\left(\lambda_{\mathrm{BS}}\right),\;\lambda_{\mathrm{BS},\min},\;\lambda_{\mathrm{BS},\max},\;\frac{Q_{\mathrm{target}}}{\lambda_{\mathrm{UE}}},\;\epsilon_{2}\right)$12:**until** $\left|u_{\mathrm{new}}-u_{\mathrm{temp}}\right|<\epsilon_{1}$ **and** $\left|\lambda_{\mathrm{BS},\mathrm{new}}-\lambda_{\mathrm{BS},\mathrm{temp}}\right|<\epsilon_{2}$13:**return** $\lambda_{\mathrm{BS},\mathrm{new}},u_{\mathrm{new}}$


## 5. Simulation Results

In this section, we validate the analysis results for the coverage probability and show the behavior and performance of the proposed algorithm through numerical simulations. All simulation results were obtained by computing the empirical coverage probability using a Monte Carlo method in a network area measuring 20×20 [km]. In every single deployment of BSs and UEs, we sampled 10,000 independent channel realizations to compute a single coverage probability conditioned on that deployment as the number of incidents received by the SINR exceeded the threshold T(K) divided by 10,000. Eventually, the marginal coverage probability $P_{c}$ was computed by taking the average of the conditional coverage probabilities of 2000 independent deployments. Unless otherwise stated, the simulation environment followed the simulation parameters in Table 1. Those simulation parameters were thoughtfully chosen based on a combination of factors, including the system model, the performance analysis, and relevant work [15,20,22].

**Figure 2:**
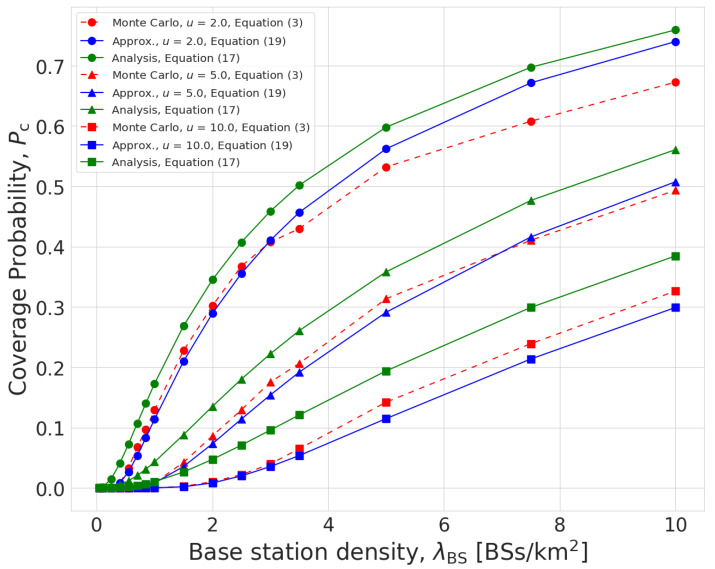
Coverage probability with respect to $\lambda_{\mathrm{BS}}$.

Figure 2 shows the coverage probabilities obtained from Monte Carlo simulation and our performance analysis. It is shown that the expressions for the coverage probability ((17) and (19)) characterized the actual coverage probability well. In particular, the approximation fn the number of in-cell users (19) did not introduce significant error on the coverage probability, and this analysis error becomes negligibly small when $\lambda_{\mathrm{BS}}\ll \lambda_{\mathrm{UE}}$. Furthermore, it is confirmed that the coverage probability is a monotonically increasing function of the BS density $\lambda_{\mathrm{BS}}$ and a monotonically decreasing function of the transmission rate *u*. This is because the SINR threshold for successful signal reception $T\left(K\right)=2^{\frac{uK}{B}}-1$ increases with the transmission rate *u*.

**Figure 3:**
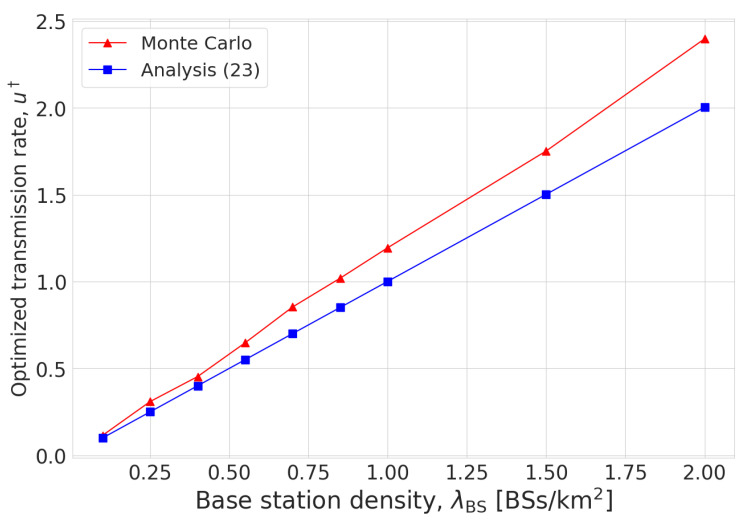
Optimized transmission rate in problem (5) for various BS densities.

Figure 3 shows the optimized transmission rates obtained from Monte Carlo simulation and the proposed method (23). It is shown that the optimized transmission rate was a monotonically increasing function of the BS density. Even though there was some error in the analysis result, it is shown to characterize the effect of BS density on the optimized transmission rate well. Furthermore, similar to Figure 2, the analysis error is shown to be negligible when the BS density was low.

**Figure 4:**
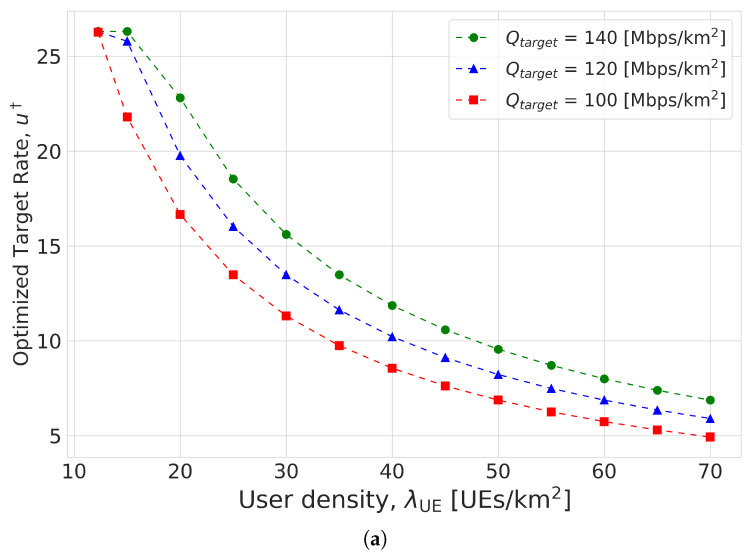

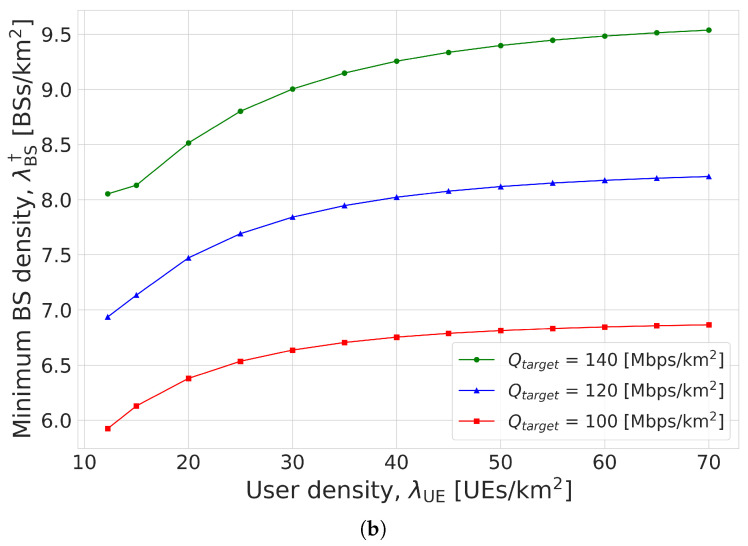
Solution to the joint optimization problem (6) for various user densities: (**a**) Optimized transmission rate. (**b**) Optimized BS density.

Based on the validations of our analysis, Figure 4 shows the results of Algorithm 2 for the joint optimization problem (6) in the second scenario. It is shown that both transmission rate and BS density increased as the performance requirement $Q_{\mathrm{target}}$ grew. In addition, if the inter-cell interference became severe with the growth in user density, the transmission rate and BS density were shown to be changed so as to increase the coverage probability. It is interesting to note that the optimized BS density was nearly saturated at a high user density, even though the transmission rate monotonically decreased with the user density. From this observation, we can see that the increase in users was mainly handled by the transmission rate control.

## 6. Conclusions

In this paper, we have investigated the wireless model aggregation for FL in small-cell networks, where BSs cooperatively aggregate the locally trained model of edge users. Based on stochastic geometry, we have analyzed the effects of geographic node deployment on the coverage probability and the model aggregation rate. With the approximation on the number of in-cell users in a typical Voronoi cell, we have derived a tractable closed form of the coverage probability in the interference-limited environment. Based on the derived expression, we have proposed two algorithms for maximizing the model aggregation rate and finding the minimum required BS density for achieving the target aggregation rate. The simulation results have confirmed that our analysis results accurately characterize the actual performance obtained using a Monte Carlo method and that the analysis error becomes negligible when the density ratio $r_{\lambda }$ is low. Furthermore, our discussions on the minimum required BS density provides insightful information for understanding the model aggregation in small-cell networks, which can be exploited as a guideline for designing networks which facilitate wireless FL.

## Figures and Tables

**Table 1:** Simulation parameters.

Symbol	Description	Value [Unit]
α	Path-loss exponent	4
*B*	Bandwidth	20 [MHz]
$\lambda_{\mathrm{UE}}$	User density	50 [users/km${}^{2}$]
*u*	Transmission rate	10 [Mbps]
*P*	Transmit power	20 [dBm]
$\sigma^{2}$	Additive noise power	−104 [dBm]

## Data Availability

All data derived from this study are presented in the article.
